# Modelling Differential Diagnosis of Febrile Diseases with Fuzzy Cognitive Map

**DOI:** 10.3390/tropicalmed8070352

**Published:** 2023-07-03

**Authors:** Okure Obot, Anietie John, Iberedem Udo, Kingsley Attai, Ekemini Johnson, Samuel Udoh, Chukwudi Nwokoro, Christie Akwaowo, Emem Dan, Uduak Umoh, Faith-Michael Uzoka

**Affiliations:** 1 Department of Computer Science, University of Uyo, Uyo 520103, Nigeria; ibibioiberedem@yahoo.com (I.U.); samueludoh@uniuyo.edu.ng (S.U.); nwokorochukwudi9@gmail.com (C.N.); uduakumoh@uniuyo.edu.ng (U.U.); 2 Department of Mathematics and Computer Science, Ritman University, Ikot Ekpene 530101, Nigeria; aniettejohn5@gmail.com (A.J.); attai.kingsley@ritmanuniversity.edu.ng (K.A.); eke5461@gmail.com (E.J.); 3 Health Systems Research Hub, University of Uyo Teaching Hospital, Uyo 520103, Nigeria; christieakwaowo@uniuyo.edu.ng (C.A.); emydan94@gmail.com (E.D.); 4 Department of Mathematics and Computing, Mount Royal University, Calgary, AB T3E 6K6, Canada; fuzoka@mtroyal.ca

**Keywords:** fuzzy cognitive map, febrile diseases, malaria, enteric fever, laser fever, yellow fever, dengue fever, HIV/AIDS, tuberculosis, urinary-tract infection, respiratory-tract infection

## Abstract

The report of the World Health Organization (WHO) about the poor accessibility of people living in low-to-middle-income countries to medical facilities and personnel has been a concern to both professionals and nonprofessionals in healthcare. This poor accessibility has led to high morbidity and mortality rates in tropical regions, especially when such a disease presents itself with confusable symptoms that are not easily differentiable by inexperienced doctors, such as those found in febrile diseases. This prompted the development of the fuzzy cognitive map (FCM) model to serve as a decision-support tool for medical health workers in the diagnosis of febrile diseases. With 2465 datasets gathered from four states in the febrile diseases-prone regions in Nigeria with the aid of 60 medical doctors, 10 of those doctors helped in weighting and fuzzifying the symptoms, which were used to generate the FCM model. Results obtained from computations to predict diagnosis results for the 2465 patients, and those diagnosed by the physicians on the field, showed an average of 87% accuracy for the 11 febrile diseases used in the study. The number of comorbidities of diseases with varying degrees of severity for most patients in the study also covary strongly with those found by the physicians in the field.

## 1. Introduction

The signs and symptoms of a disease distinguish one disease from another. Sometimes, these signs and symptoms are so similar that it becomes a challenge to make a fast and accurate distinction and this could result in an inaccurate diagnosis. Since diagnosis is the bedrock of medical practice [1], an inaccurate diagnosis could lead to complications, and, if not handled properly, could lead to the death of the victim [2]. Febrile diseases are fever-based diseases with similar and overlapping symptoms that are often confusable and difficult to differentiate. They are prevalent in tropical and subtropical regions where climatic conditions such as temperature, humidity, and evaporation contribute immensely to promoting the spread. According to Attai et al. [3], tropical locations around the world are severely affected by infectious diseases.

The knowledge of the symptoms, the etiology of a disease, and the thought process gained during practice help a physician to associate symptoms with a disease. The cognitive mapping operations could be transferred into a machine for more accurate and faster processing than a human physician does. Among many other challenges, the traditional logic of a computer does not support human reasoning as it exhibits exactness in its methodology [4]. This shortcoming of conventional logic becomes more pronounced in medical diagnosis because of ambiguities associated with a patient’s medical history, laboratory investigation results, symptom elicitation, etc.

The limitation of conventional logic is overcome with fuzzy logic technology capable of resolving ambiguities and uncertainties through collaboration and aggregation and reasoning with approximation as done by the human physician [5]. With fuzzy logic, transferring the knowledge of the human physician becomes easier as the cognition operation can be mapped with fuzzified datasets and later defuzzified into crisp outputs.

Physicians are prone to errors, and medical diagnostics errors could be life threatening [2]. The errors could be because of a lack of experience, the large volume of data due to an influx of patients requiring services from a limited number of physicians, poor accessibility to patients’ previous records to obtain medical history, the inability of patients to express their feelings of a particular symptom, among other reasons. They (physicians), therefore, need a tool that can assist them in reducing these errors. One such tool is the medical decision support system (MDSS), which has been useful in making critical decisions.

One of the main reasons the population, particularly those in tropical regions, cannot get medical care, according to the World Health Organization (WHO), is a lack of medical personnel. According to Mehta et al. [6], African healthcare facilities are vastly understaffed and under-resourced. The WHO [7] report of the 2018 accessibility to medical personnel in Africa is appalling, as the density ratio of 5000 patients is equated to 1 physician and 6 nurses. These statistics justify the need for a decision-support diagnostic tool to aid in curbing the rising cases of mortality caused by, among other factors, the lack of access to medical facilities by an average person living in tropical regions, especially in rural settings and resource-scarce areas.

The study aims at developing an FCM-based MDSS for febrile diseases to solve the problems of (i) poor access to medical care for febrile diseases patients in low-to-middle-income countries and resource-scarce areas and (ii) poor differentiation of signs and symptoms of febrile diseases by inexperienced physicians. The objectives of the study are to (a) gather datasets from patients of febrile diseases in four states in Nigeria where febrile diseases are prevalent, (b) obtain experiential knowledge of medical doctors who specialize in febrile diseases, (c) develop an FCM-based model for the differential diagnosis of 11 febrile diseases, (d) analyze the results obtained from the developed system and compare with the results of the domain experts, and (e) evaluate the number of comorbidity of diseases. The uniqueness of this study is in (i) the method of generating weights for the adjacency matrix, (ii) the inclusion of a decision filter to capture the emotional feelings of the patients to form part of the decision variables, and (iii) the evaluation of comorbidity of diseases, which is known to be one of the causes of mortality because of inadequate attention given to patients in the course of diagnosis.

The rest of the paper is organized as follows; Section 2 presents the related literature while Section 3 presents the methodology of carrying out the research. The results of the experiment carried out are presented and analysed in Section 4. Recommendations made and conclusions drawn are presented in Section 5.

## 2. Related Literature

A disease associated with fever is commonly referred to as a febrile disease. Prasad et al. [8] demonstrated a wide range of pathogens associated with several febrile diseases. However, the distribution of the disease varies by geography, season, age, and immunity of the patient. According to Bell [9], the relative frequency of acute febrile syndrome varies widely with geography, living condition, and occupational exposure. There has been some research on the differential diagnosis of confusable symptoms of febrile diseases. Malaria tends to become the default diagnosis of febrile diseases due to its ubiquity and severity [10,11], such that if a patient presenting the symptoms is tested for malaria and the result is found to be negative, such a patient is left untreated for other diseases by an inexperienced medical doctor with the risk of complications. According to Crump et al. [12], healthcare workers often lack epidemiological information or the laboratory services necessary to support rational diagnostic and management decisions for patients with negative malaria diagnostic test results.

In order to treat a nonmalaria febrile illness properly (keeping in mind that patients may have malaria concurrently with another disease, especially in high endemic areas), the pathogens that cause a febrile disease must be known. If the agent is not identified, knowing the category of the pathogen (parasitic, bacterial, or viral) is useful for deciding on treatment [13]. This requires high-level accuracy of differentiation of the symptoms. Patients with enteric fever develop problems and may require therapy with longer antibi-otics to remove the infection. Enteric fever symptoms include fever, diarrhoea, muscle aches, stomach pain, rash, and others; therefore, certain guidelines are important to assist clinicians in performing the right tests and treating patients with enteric fever [14]. The ability to detect Mycobacterium tuberculosis (MTB) infection, recognize the factors that lead to tuberculosis (TB) disease, receive preventative therapy, and put methods in place to track infections and treatment completion all contribute to better control of tuberculosis [15]. Dengue fever is the most prevalent viral illness spread by mosquitoes; although it is typically moderate, dengue fever can progress into a severe type that can be fatal [16].

A review by the WHO [17] on the different tools used to evaluate acute febrile illness (AFI) in South India shows malaria to be the commonest cause of AFI, followed by dengue, scrub typhus, bacteremia, and leptospirosis. It was also revealed that malaria diagnosed by smear microscopy was more popular than other methods of tests.

Considerable research is undertaken on the alternative diagnostic methods for malaria, tuberculosis, HIV/AIDS, and dengue fever, leaving the other febrile diseases almost neglected. The effect of this is positively felt in malaria, where there was about a 40% reduction in the incidence of malaria between 2000 and 2015 [7]. A significant challenge is the acute shortage of physicians in febrile disease-prone areas. The WHO [7] gave the 2018 report on accessibility to medical personnel in Africa. According to the report, the density ratio of a physician to a 5000 populace is 1, while that of nurses/midwives is 6. This poor accessibility has affected the proper diagnosis and treatment of febrile diseases, thereby increasing the morbidity and mortality rate. The experts have a great role to play in developing systems capable of retaining knowledge and assisting them in their jobs. The medical decision support system (MDSS) has been found useful to medical practitioners in an attempt to increase the accessibility of patients to medical care and reduce the workload of personnel.

Although several approaches are used to enhance processes of improving individual health, the introduction of the fuzzy logic approach seems more human-like because of its ability to deal with uncertainty and ambiguity, which are recurring attributes in medical records. Das et al. [18] adopted fuzzy logic to model doctors’/medical experts’ confidence levels in diagnosing diseases in the patient. Their method is composed of the following four steps: (i) the modelling of the antecedent part of the rules, which consists of linguistic assessments of the patient’s symptoms provided by the doctors/medical experts with their corresponding confidence levels by using generalized fuzzy numbers; (ii) the modelling of a consequent part, which reveals the degree of association and the degree of non-association of diseases into the patient, by using intuitionistic fuzzy system (IFS); (iii) the use of an IFS aggregation operator in the inference process; and (iv) the application of a relative closeness function to find the final crisp output for a given diagnosis. Nilashi et al. [19] proposed a knowledge-based system for breast-cancer classification using fuzzy logic to assist medical practitioners in their clinical decision support towards their healthcare practice. The proposed knowledge-based system proves to have a better prediction accuracy (0.932) for breast cancer in relation to PCA-SVM (0.867), PCA-KNN (0.823), and decision tree (0.929).

Amjad et al. [20] employed an expert soft sets system (SES) based on the soft sets and the fuzzy set theory to diagnose dengue fever. They calculated the risk percentage of 30 patients with the help of soft sets, and it was noted that 13 patients were suffering from dengue while the other 17 patients had no complaints of dengue fever. Sharma et al. [21] introduced the concept of mediative fuzzy relation between the conventional fuzzy set and the intuitionistic fuzzy set. The mediative fuzzy projection was used in the diagnosis of COVID-19 in post-COVID-19 patients. The results obtained from the study were compared with that of conventional and intuitionist fuzzy projection and found to covary strongly. Magwili et al. [22] provided a preliminary diagnosis for patients suffering from mosquito-borne diseases by comparing the system’s preliminary diagnosis with the expert’s diagnosis in a total of 80 tests with 20 tests per disease; 71.67%, 83.33%, and 91.67% of the time, the system correctly prediagnosed dengue, chikungunya, and malaria, respectively. For other diseases, the system correctly identified the unlikelihood of having the said mosquito-borne diseases 91.67% of the time. Moreover, a chi-square test was also conducted with a level of significance of 0.05, yielding a *p*-value of 0.464. According to Putra and Prihatini [23], tropical infectious diseases require appropriate treatment with the active participation of a doctor and patients. In their result for defuzzification, they calculated the sequential and combined certainty factor, which represents the belief percentage of disease diagnosis suffered by the patient. The results of the expert diagnosis with the expert system for the given cases indicate the system has similarity diagnosis with the expert at 93.99%.

Ekong et al. [24] demonstrated that information technology and medicine could successfully operate together using differential diagnosis by applying fuzzy logic to medical informatics. The result increased productivity in the grid system by an average of 20%. They suggested the need to apply fuzzy logic because it will help to resolve conflicts that may arise from ambiguity, uncertainty, and imprecision in the investigation of tropical diseases. A fuzzy cognitive map (FCM) is a technique for realizing an efficient MDSS. It is built based on the experience of the domain experts who provide the degree of influence and causal knowledge of one concept to another. This means it relies on what an expert, such as a physician, perceives as the causal relationship of a symptom, such as a headache, to a disease such as malaria. This degree of influence is captured and represented as a link between headaches and malaria. According to Bourgani et al. [25], a fuzzy cognitive map is a soft computing technique used for causal knowledge acquisition and supporting the causal knowledge reasoning process. The FCM modelling approach resembles human reasoning; it relies on the human’s expert knowledge of a domain, making associations along generalized relationships between domain descriptors. Bourgani showed different forms of FCM structures for MDSS, made comparisons, and recommended temporal concepts to be included in the design of MDSS for dynamism and efficiency.

Amirkhani et al. [26] identified the different FCM structures used in MDSS after a thorough analysis of each structure and reviewed various diagnoses and decision-support problems addressed by FCMs to determine their contributions to improving medical diagnosis and treatment. Groumpos [27] explored the concept of causality to model a new state space, advanced fuzzy cognitive map (AFCM) methodology for modelling COVID-19 diagnosis. He noted that correlation does not imply causality while causality always implies correlation. He found that the FCM theories are probably the only ones that explore the causality between the variables of medical problems in a sound mathematical and scientific foundation. In Papageorgiou et al. [28] the diagnosis of the degree of severity of pulmonary infection using 33 symptoms of infectious diseases was carried out using the FCM technique. Hypothetical cases were used for the simulation of the results, showing the calculated severity of pulmonary infection to be above 90%. FCM Expert, a software for FCM modelling, was used to analyze a scenario and perform pattern classification [29].

Mpelogianni and Groumpos [30] modified the conventional FCM to obtain a mathematical model that uses a state-space approach to disaggregate the concepts into state concepts, input concepts, and output concepts. The model was then used to compute a building’s energy consumption and management of its loads. Results of computations when compared with that of the conventional FCM were found to be more accurate. According to Apostolopoulos and Groumpos [31], FCMs are potentially trustworthy because they incorporate human knowledge. Based on the parameters of trust, transparency, and causality, an explainable AI is proposed for FCM-based systems.

The architecture and features of the software were shown and discussed, including the characteristics, such as its ability to improve system convergence. A case study of FCM-based classification for modelling the resistance of HIV-1 mutations was demonstrated using a particle-swarm optimizer. A differential diagnosis of 6 eye diseases with 23 symptoms was undertaken by Obot et al. [32], where 2 independent opticians diagnosed 20 patients each and compared with the results of diagnosis using FCM with the Hebbian learning rule. The results show 65% and 45% accuracy for the first and second opticians’ diagnoses, respectively. Apostolopoulos et al. [33] developed a state space advanced FCM to detect Coronary Artery Disease (CAD). The state space concepts consist of the input concepts, the state concepts, and the output concepts, where the state concepts depict the concepts that describe the operations of the system. This was later embedded in diagnostic rules developed by cardiologists. A total of 303 patient datasets collected from the Department of Nuclear Medicine of Patras, in Greece, were used to train and test the developed system. The results, compared with the classical FCM, showed 85.47% accuracy, which is a 7% higher accuracy than the conventional method of diagnosis. Apostolopoulos and Groumpos [34] solved the problems of ambiguity and uncertainty in coronary artery disease diagnosis using the noninvasive method with FCM. The results obtained showed an accuracy of 78.2%, which were reported to be better than what was obtained from other algorithms.

A time unit proposed by Bourgani et al. [35] that can follow disease progression is introduced into FCM to develop a diagnostic tool for differential diagnosis of pulmonary diseases (acute bronchitis and common-acquired pneumonia). Time-based FCM was proposed here because the values of weights and concepts of such diseases change according to the time interval. Uzoka et al. [36] proposed a framework for differential diagnosis of tropical confusable diseases using a fuzzy cognitive-map engine where 11 symptoms of 7 diseases were found to be confusable. The study employed the experiential knowledge of practising physicians and utilized a brute-force algorithmic procedure to mimic the mental algorithm used by physicians in the diagnosis process. A case study of malaria was carried out with 20 datasets, of which 55% matched the physicians’ diagnoses, and 85% matched the FCM diagnoses. Uzoka et al. [37] showed a higher (though equally significant) correlation between the FCM results and actual diagnosis (AD), and between initial hypotheses (IH) and AD. The comparative summary showed that the IH by the physicians correctly matched the final diagnosis in 55% of the cases, whereas AD of the FCM was 85%. This also connotes that the correlation between the physician’s initial hypothesis and the FCM diagnosis was not significant.

Hoyos et al. [38] used fuzzy cognitive maps to enhance clinical decision-support systems for dengue fever. The developed model showed a good classification performance with 89.4% accuracy and could evaluate the behaviour of clinical and laboratory variables related to dengue severity (it is an explainable method). Their model serves as a diagnostic aid for dengue that could be used by medical professionals in clinical settings and [39] applied a fuzzy cognitive map for geospatial dengue outbreak-risk prediction in tropical regions of Southern India. The accuracy of the proposed FCM-based classification approach is much better than the benchmark machine-learning algorithms, which show a deficiency in working with small datasets and without being able to use experts’ knowledge.

## 3. Methodology

**Figure 1:**
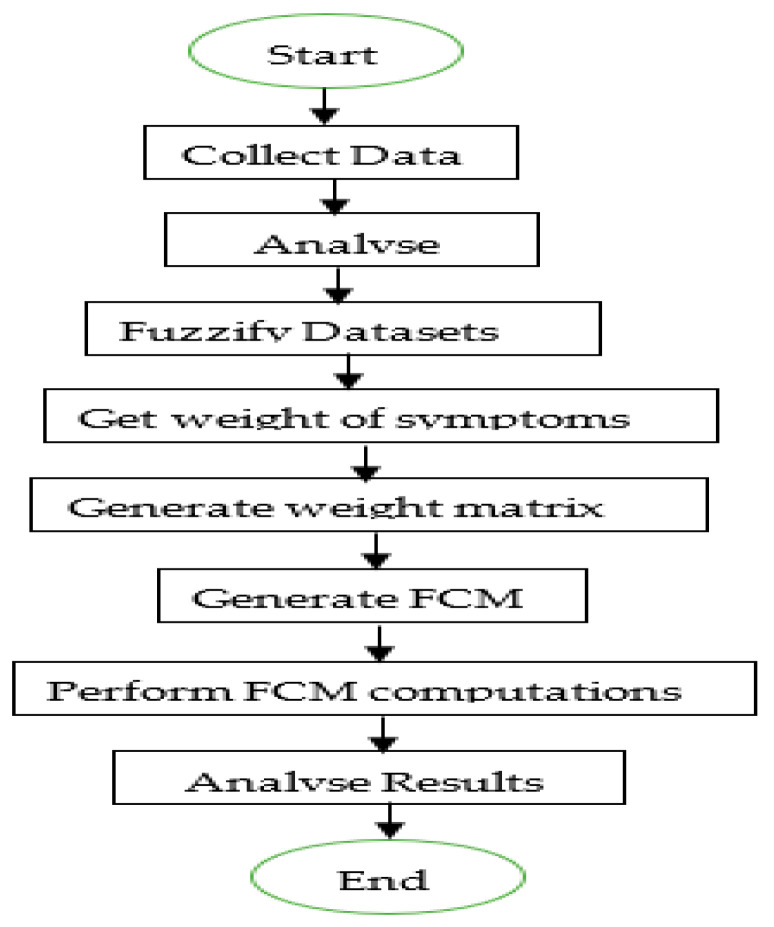
Flowchart of the system methodology.

The study started with the process of collecting datasets from which they were fuzzified and analyzed to obtain weights of each symptom and their corresponding diseases. The weights obtained were validated by the medical team of the project and used to generate a weight matrix and a fuzzy cognitive map. The training was then carried out using the Hebbian learning rule on the sigmoidal activation function. The results obtained were analysed using the confusion matrix to perform binary classification. The flow chart in Figure 1 summarizes the steps employed in carrying out the research. Each of the steps is discussed subsequently.

### 3.1. Data Collection

Febrile disease datasets were collected through a field study in four (4) states in the Niger Delta region of Nigeria, comprising; Akwa Ibom, Cross River, Rivers, and Imo state. An Open Data Kit app [40] was used to collect the datasets based on two questionnaire instruments validated by domain experts (medical doctors). One data source extracted experiential knowledge from a random sample of sixty-two (62) physicians in the study area, in private and public health facilities, who have expertise in diagnosing febrile diseases. The second data source was gathered from a patient-consultation instrument designed to assist physicians in eliciting patients’ symptoms and recording preliminary diagnoses, including further investigations and final diagnoses outcomes. The second data source was employed in this research. Each symptom was rated on a scale of 1–6, representing absent, very low, low, moderate, high, and very high severity, respectively. The final diagnosis for each patient examined by the medical doctor was also rated on the same scale. In concluding the final diagnosis, the physicians also were cognizant of the risk factors associated with each of the eleven (11) febrile diseases considered in the study. These risk factors provide physicians with the opportunity to associate them (e.g., genetic conditions, high blood pressure, high cholesterol level, exposure to mosquito bites, and travel to endemic regions) with each disease under consideration on a numeric scale (1 = No effect, 2 = Weak effect, 3 = Moderate effect, 4 = Strong effect, 5 = Very strong effect).

The dataset used for the study contains 4879 patient records out of which 1617 were found to contain some missing fields so were removed. To preserve the dataset’s integrity, records with omitted symptoms or diseases were removed. After this, of the 3253 records left, 185 were found to be that of children below 5 years of age and the data collection instrument did not capture some of the symptoms presented by patients with tropical febrile conditions of this age range. The age at which a person transitions from childhood to adulthood may vary depending on the culture, and the legal definition often ranges from 16 to 21 years [41]. In this study, patient records above 16 years were selected. The records of those above 16 years numbering 2465 were therefore tagged as the adult datasets and used for this study.

### 3.2. Fuzzification of Datasets

The values in the datasets collected were crisps and needed to be fuzzified. This was done with the triangular membership function given by the formula.

(1) $$F\left(x\right)=\frac{x-0.5}{6}$$

(2) $$F\left(x\right)=\{\begin{array}{c} 0.0<=x<=0.1\ldots \ldots \ldots \ldots \ldots \ldots \ldots ..No\;Disease\\ 0.1<=x<=0.4\ldots \ldots \ldots \ldots \ldots \ldots \ldots ..VeryLow\\ 0.35<=x<=0.5\ldots \ldots \ldots \ldots \ldots \ldots \ldots Low\\ 0.40<=x<=0.60\ldots \ldots \ldots \ldots \ldots \ldots .Moderate\\ 0.55<=x<=0.80\ldots \ldots \ldots \ldots \ldots \ldots .High\\ 0.80<=x<=1.0\ldots \ldots \ldots \ldots \ldots \ldots \ldots VeryHigh \end{array}$$

The choice of the triangular membership function stems from the fact that the boundary between linguistic variables in the datasets is not so thin to warrant the use of other membership functions, such as trapezoidal or Gaussian. Again, a study by Princy and Dhenakara [42] shows that the triangular membership function gives better accuracy on medical datasets.

### 3.3. Weight of Symptoms

Based on the frequency of reported cases of a symptom on the entire datasets obtained, the Pearson, Kendall, and Spearman correlation tools were used to determine the correlation between each disease and the corresponding symptom. The average value of the three tools was obtained and used to rank the symptoms accordingly. Outliers were removed, and a threshold was determined by a team of 10 medical doctors after observing the value of each symptom and its rank. For example, 13 symptoms were identified as symptoms of malaria with their corresponding weights as shown in Table 1. Bitter taste in the mouth (BITAIM) was observed to rank first with a value of 0.52, followed by chill and rigour (CHLNRIG) with 0.39. Vomiting (VMT) was ranked last with a weight value of 0.19 and used as the threshold weight for malaria. The listing of the symptoms and their ranking results for other diseases are presented in Appendix A.

**Figure 2:**
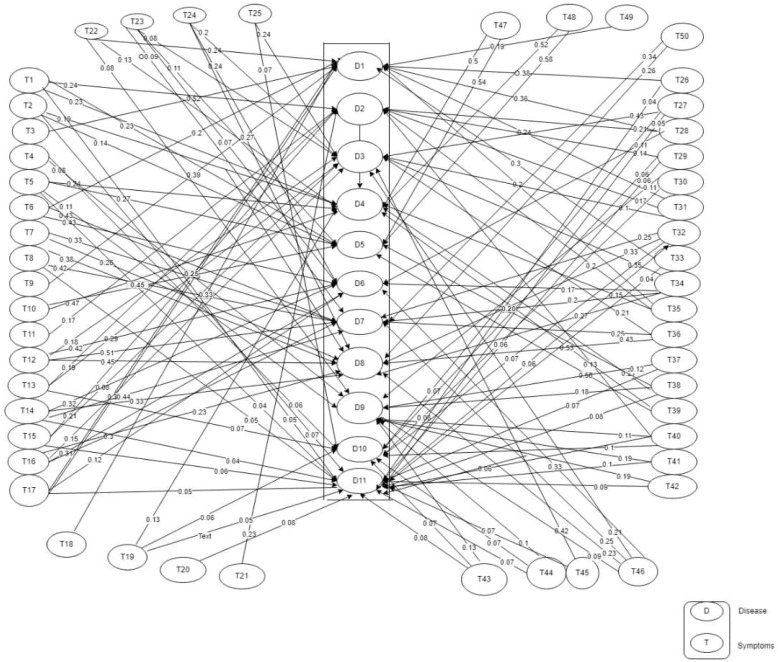
Fuzzy Cognitive Map (FCM).

All these were combined to generate a weight matrix which was used to draw the fuzzy cognitive map, as shown in Figure 2.

The causal relationship between the symptoms of a particular disease and the disease is shown on a fuzzy cognitive map (FCM). A synergy is established amongst the eleven diseases identified as febrile due to their similarity in symptoms and the aetiology of the disease. The value attached between each of the symptoms and its corresponding disease represents the weight of the symptom to the disease and, therefore, its causal value. The concepts (symptoms and diseases) for the differential diagnosis of the eleven diseases are shown in Appendix B. There are 61 concepts comprising 50 class concepts (symptoms), labelled T1 to T50, and 11 decision concepts (diseases), labelled D1 to D11. The corresponding map (FCM) is shown in Figure 2.

### 3.4. The System Architecture

**Figure 3:**
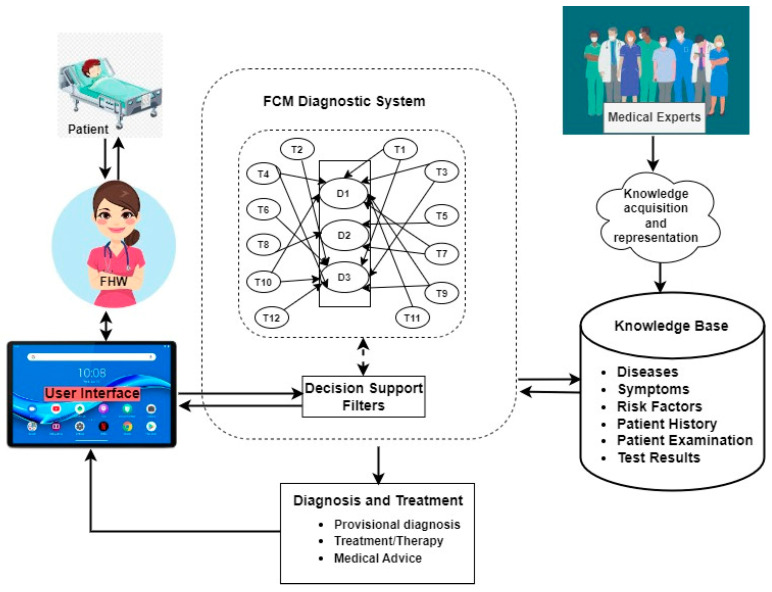
System architecture of FCM-based medical DSS for febrile disease diagnosis.

The components of the system interact with one another, as depicted in the architectural design in Figure 3, which comprises the medical experts, the frontline healthcare workers (FHWs), the patients, the knowledge base, the diagnostic engine, the FCM engine, the decision-support filters, and the graphic user interface as the main components. A patient’s signs, symptoms, and laboratory test results are captured into the knowledge base through the graphic user interface by the FHWs. This information is stored in the knowledge base of the system along with static and dynamic (experiential) knowledge of the medical experts for later use by the diagnostic engine of the system for processing. The risk factors and emotional feelings of a patient are captured through the decision support filters to the diagnostic engine. The knowledge base feeds the diagnostic engine, which first fuzzifies the datasets and then maps the corresponding signs and symptoms with the appropriate diseases. The computed values are thereafter defuzzified into crisp outputs and sent back to the FHWs as the diagnostic results.

### 3.5. FCM Computations

The value of each concept is influenced by the value of the connected concepts with the corresponding causal weights and their previous value. The value of concept represents the degree of severity of a particular symptom of a disease as assigned by the medical doctor during the diagnosis. The causal weights are generally agreed by the 10 medical doctors to be the causal effects or the degree of influence on the corresponding symptom. The model used for the computations is given as:

(3) $$A_{i}\left(t+1\right)=f\left(A_{i}\left(t\right)+\underset{j\neq i,j=1}{\sum }(A_{j}\left(t\right).E_{\mathrm{ji}}\right))$$

where $A_{i}\left(t+1\right)$ is the value of concept $C_{i}$ at step (t+1); $A_{j}\left(t\right)$ is the value of concept $C_{j}$ at step t, $E_{\mathrm{ji}}$ is the weight of the interconnection from $C_{j}$ to $C_{i}$, and f is the threshold function. A concept cannot be linked to itself, so j<>i(j is not equal to i), the subscripts (j and i) denote the position of the concept with i being the position of the source concept and j the position of the destination concept; for example, if fever is concept 5 (C_5_) and malaria is concept 51(C_51_), then i = 5, j = 51. A link between fever and malaria is C_ij_ and the weight (value) is established between the link as the causal effect. In our map, T1–T50 represent C1–C50 and D1–D11 represent C51–C61. Each of the 61 concepts takes 6 fuzzy values of “no disease, very mild, mild, moderate, high, and very high” membership functions. In computing the final value that represents the diagnostic state of a patient, the initial vector that shows the state of health of a patient, as expressed by the physician that interrogated the patient, is multiplied by the weight matrix following Equation (3). The result is applied to the sigmoidal continuous function to obtain the final diagnosis. It was at the sixth iteration that equilibrium was reached. The symptoms recorded for patient number Pat_1261 as shown in Table 2 are used to compute the scenarios in Table 3 and Table 4.

Ignoring symptoms that do not result in malaria for patient number Pat_1206, the initial vector is shown as the first row of Table 3 while the corresponding weight is represented in the second row of the table. Applying Equation (3), the results are generated until an equilibrium is found at the sixth iteration and the results are used to find the final diagnosis using the signum continuous activation function $f\left(x\right)=1/1+e^{-x}$. This gives 0.62 after the 6th iteration. The living condition and work habits of the patient were found to place the patient at risk of malaria, accounting for 0.17. The final diagnostic value of the patient is therefore put at 0.790.

Scenario 2: from the symptoms presented in Table 2, the symptoms associated with enteric fever are SWRFVR (0.75), HDACH (0.75), and LTG (0.58) and their weights are 0.23, 0.21, and 0.17, respectively. The computations for the diagnosis of enteric fever for PAT_No 1261 are shown in Table 4. After the 6th iteration, the signum activation is computed to give a diagnosis of 0.75 and the patient was not found to be at risk of enteric fever. The patient showed positive emotions that accorded about 5% (0.05) chance of not suffering from the disease, as computed by the FCM

Table 5 shows the final diagnosis as computed for patient number Pat_1261 for malaria, enteric fever, and other diseases. This shows the patient has comorbidity of malaria, enteric fever, upper respiratory-tract infection (URTI), lower respiratory-tract infection (LRTI), and tuberculosis (TB) diseases with varying degrees of severity. Equation (4) is used for the classification of the results.

(4) $$F\left(x\right)=\{\begin{array}{c} 0.3<=x<=0.4\ldots \ldots \ldots \ldots \ldots \ldots \ldots No\;Disease\\ 0.4<=x<=0.5\ldots \ldots \ldots \ldots \ldots \ldots \ldots Very\;mild\\ 0.5<=x<=0.6\ldots \ldots \ldots \ldots \ldots \ldots \ldots Mild\\ 0.6<=x<=0.7\ldots \ldots \ldots \ldots \ldots \ldots \ldots Moderate\\ 0.7<=x<=0.8\ldots \ldots \ldots \ldots \ldots \ldots \ldots High\\ 0.8<=x><=1.0\ldots \ldots \ldots \ldots \ldots \ldots very\;High \end{array}$$

## 4. Results and Discussion

### 4.1. Results

A sample result of the study is presented in Appendix C. It comprises 50 of the 2465 patients’ results of the datasets diagnosed by the domain experts and the computational results as processed by FCM.

### 4.2. Discussion

**Figure 4:**
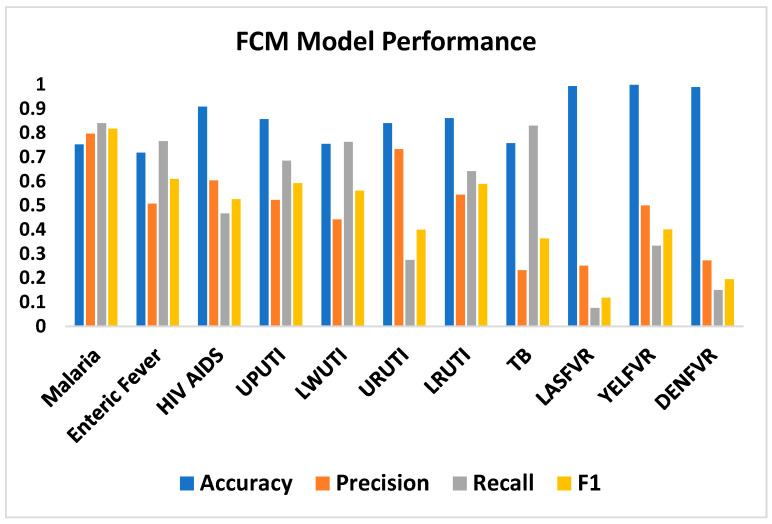
FCM performance evaluation.

A binary classification of the results into patients diagnosed with a specific disease and those not diagnosed was carried out on the actual and predicted (computed) results. The performance evaluation of the 11 diseases is shown in Figure 4 and Table 6. From the results of these metrics, the true positive (TP), true negative (TN), false negative (FN), and true negative (TN) values were extracted, as shown in Table 7, while Table 8 presents the actual and predicted diagnosis for each disease. The average accuracies of 87%, precision of 53%, recall of 50%, and F1 of 51% were recorded. Four diseases (LWUTI, TB, LASFVR, and DENFVR) performed below 50% precision measure, while URUTI, LASFVR, YELFVR, and DENFVR performed below 50% of recall and the F1 measure had URUTI, TB, LASFVR, YELFVR, and DENFVR below 50%. Malaria has the highest number of diagnoses, of 1631 actual diagnostic results and 1721 predicted diagnoses. This is followed by enteric fever with 710 actual and 1072 predicted diagnoses.

There are fewer cases of predicted diagnoses than actual cases in HIVAD (270 actual and 209 predicted), URTI (478 actual and 179 predicted), LASFVR (13 actual and 4 predicted), and YELFVR (2 actual and 1 predicted), while DENFVR has an equal number (11) of actual and predicted results. The results of the number of diagnoses per disease are presented in Table 7. A total of 1050 patients representing 43% of the diagnoses have a comorbidity of between two and seven diseases. It was noticed that in most of the comorbidity of five to seven diseases, HIVAD, TB and MALARIA, were found in 79% of the comorbidities. Comorbidity on the actual results covary strongly with that of the predicted results. For instance, while patient number 14 suffered from six diseases in the predicted results, seven disease results, including HIVAD and TB, were found for the same patient in the predicted results.

### 4.3. The Implication of the Results

Results of the computations show that malaria is ranked first among the 11 diseases in terms of prevalence. This confirms a study by Crump et al. [12], which shows malaria as a default disease in tropical regions. The study has also revealed that malaria is comorbid to other diseases, with 830 cases representing 79%, indicating that most malaria patients need to be investigated for other diseases. According to Crump et al. [12], patients found to be negative for malaria tests are left unattended with the belief of wellbeing. That a patient is not positive for malaria does not suggest that the patient cannot suffer from other diseases. An FCM-based application can be used by the FHWs to diagnose febrile diseases, thereby curbing the problem of acute shortage of medical doctors, especially in rural settings [7]. This implies a life-saving measure for patients who resort to self-help because of delays in attending to them. Medical doctors will be relieved of the stress they undergo due to a large number of patients waiting to consult them on a daily basis. Such situations sometimes lead to wrong diagnoses, especially with overlapped symptoms that could be very difficult to differentiate by an inexperienced physician. According to Keller et al. [43], tropical diseases such as malaria, which are regarded as regional diseases, are increasingly encountered in the developed world due to travelers. There is a dearth of specialists in tropical diseases in the developed world; therefore, travelers from tropical regions are prone to these diseases but lack access to medical treatment. This gap could be bridged with an intelligent-decision support tool which such travellers will rely on for diagnosis and treatment of such diseases.

Ethical and policy implications are vital aspects of research that researchers should carefully consider. According to Goodman [44], making an accurate diagnosis is necessary for many more reasons than just the personal satisfaction that comes from being correct. It is based on the fact that accurate diagnoses have better outcomes more often than faulty diagnoses do. It is also predicated on the negative consequences that mistakes bring forth. Currently, there is an abuse of treatment of febrile diseases due to self-help carried out by patients. Misdiagnosis and inaccurate diagnosis are practised by drug vendors without following laid-down procedures for diagnosis and therapy. MDSS based on FCM has the potential to assist medical doctors, nurses, FHWs, and patients with some ethical concerns. MDSSs play a significant role in promoting an efficient and effective healthcare system [45]. To this end, a number of ethical issues that follow the use of intelligent machine diagnoses were followed in the process of data collection, planning, design, and development of this study. Computers are not meant to usurp the work of medical experts; the study is designed to aid experts through FHWs in taking the final decision on the state of wellbeing of a patient before a therapy decision is taken. As part of the work ethics and practice, the FHWs are the end users of the system and not the patient. The laid down procedures and standard guidelines accepted by the medical profession are used in implementing the study. The vulnerability and gender of a patient are respected and given due recognition from data gathering to modelling and the same goes for privacy and trust, which are also ensured. As the standards are revised, the MDSS is also revised with the responsibility of appropriate use of the system to optimize the ethical concerns to encourage the users. Adequate training of the FHWs on the use of an MDSS that results from this study to enhance its usage is part of ethics regulation. This is to ensure that the MDSS is used appropriately with no intention of abuse and to establish a lasting relationship between the developers and the medical practitioners. The relationship is meant to promote working ethics through thorough scrutiny of the datasets, procedures, rules, and standards that go into the development of the system [46]. This makes the development process both client and problem centred, using the Agile software engineering methodology [47]. The Beauchamp and Childress [48] principle of beneficence and nonmaleficence is employed to ensure the wellbeing of patients is maximized by assisting decision-makers as much as possible to deliver healthcare without let or hindrance. The certificate number for the study’s ethical approval is CRSMOH/RP/REC/2022/357.

## 5. Conclusions

The explicit knowledge embedded in fuzzy systems and the implicit knowledge in the neural networks are mapped cognitively to form a fuzzy cognitive map. FCM system provides a learning capability to adjust expert (physicians’) knowledge and automatically generate additional fuzzy rules and membership functions to aid in the diagnosis of a disease. FCM utilizes machine-learning algorithms to model a system. It models a system characterized by uncertainty, imprecision, causality, and complexity as found in medical diagnosis. These uncertainties and causalities are expressed in linguistic terms, which depict a causal relation between concepts where concepts are entities used in modelling FCM. Each concept represents a link to another concept with a degree of influence of a source concept to a destination concept.

In this study, 11 diseases and 50 symptoms represent the FCM concepts. The symptoms of the 11 febrile diseases were gathered from 3253 patients with febrile diseases. A total of 2465 of these are those 16 years and above whose records were used for the study. With the diagnoses done on these records by 62 physicians specializing in febrile diseases, the classification of the records was done in 11 clusters where each cluster represents each disease. FCM was then employed to mimic the physician’s diagnoses after the weight of the link between each of the source and destination concepts was determined and ascertained by 10 physicians. With the weights and the degree of symptoms for each patient, the values were fuzzified for the FCM using the Hebbian learning rule employed to determine the diagnostic value of the 2465 patients. Results obtained were compared with those obtained by the experts and found to covary positively.

Binary classification of the computed results was done using the confusion matrix, the results of the classification show an average accuracy of 87%, while the precision, recall, and F1 performance indicators had an average of 50%, respectively. One startling revelation of this study is the amount of comorbidity of the diseases in so many patients. A total of 1050 patients were found to have comorbidities ranging from two to seven diseases. Most of the comorbidities that are above four diseases had malaria, HIVAD and TB among the diseases. We hope to find the reasons behind these clusters of diseases in a patient in our future research. The use of decision-support filters to capture risk factors such as environmental factors and living conditions of a patient and emotional factors such as sadness, happiness, anxiety etc., was noticed as a gap in the literature which has been filled in this study.

These factors embedded in our study will help the system in mimicking the human-like reasoning of an expert while interrogating a patient, especially with the cognitive ability of FCM. A study by Keller et al. [43] shows that emotions such as anger, fear, boredom, etc., can lead to stress that is capable of increasing the degree of influence on a symptom of a disease. As a corollary, positive emotions such as love, happiness, success etc. can reduce stress and help to reduce the influence of a symptom. With a trustworthy reservoir of data and diagnostic results obtained from this study, a case-based reasoning (CBR) diagnostic methodology hybridized with FCM is suggested for further study to improve the results obtained here.

The imbalance of the datasets with malaria and enteric fever shows more than 80% of the entire datasets while yellow fever, dengue fever, and laser fever together form about 2% of the datasets is of concern. This imbalance would have contributed to the results not being so good. The early convergence of FCM is also a weakness of the study as can be seen in the equilibrium being reached at as low as six iterations in some cases. Further study is proposed with the exclusion of yellow fever, dengue fever, and laser fever. In the future, the research would be extended to interval type-2 and intuitionistic fuzzy logic.

The results of this study have been accepted by our team of medical doctors after evaluation but with a recommendation for parallel implementation with the conventional system of diagnosis. This is more so, given the fact that the app developed from the result is meant to be operated by FWHs and not the patients.

## Figures and Tables

**Table 1:** Symptoms rankings for malaria.

SN	MAL Symptoms	Pearson Rank	Kendall Rank	Spearman Rank	Mean
1	BITAIM	0.528	0.477	0.545	0.52
2	CHLNRIG	0.391	0.368	0.422	0.39
3	GENBDYPN	0.381	0.349	0.404	0.38
4	HDACH	0.372	0.324	0.378	0.36
5	FVR	0.339	0.303	0.343	0.33
6	HGGDFVR	0.318	0.284	0.327	0.31
7	MSCBDYPN	0.297	0.273	0.316	0.30
8	FTG	0.251	0.233	0.272	0.25
9	SUDONFVR	0.252	0.218	0.250	0.24
10	LTG	0.245	0.218	0.252	0.24
11	CTRH	0.194	0.188	0.215	0.20
12	NUS	0.185	0.190	0.219	0.20
13	VMT	0.187	0.180	0.207	0.19

**Table 2:** Symptoms Reported by Patient Number Pat_1261.

Symptom	Degree of Severity
CTRH	0.75
CHSP	0.58
CGHDRY	0.75
DIFBRT	0.58
DRYCGH	0.75
FVR	0.41
HGPSFVR	0.75
HGGDFVR	0.75
SWRFVR	0.75
SUNDONF	0.75
GENBDYP	0.58
HDACH	0.75
LTG	0.58
STRTRT	0.75

**Table 3:** Scenario 1 for Pat_1261 computations for Malaria.

Initial Vector	0.75	0.41	0.75	0.75	0.58	0.75	0.58	A_i_(t + 1)	Signum Function
Weight	0.2000	0.3300	0.3100	0.2400	0.3800	0.3600	0.2400	-	-
1st iteration	0.1500	0.1353	0.2325	0.1800	0.2204	0.2700	0.1392	2.327	0.688
2nd iteration	0.0300	0.0446	0.0721	0.0432	0.0838	0.0972	0.0334	1.404	0.646
3rd iteration	0.0060	0.0147	0.0223	0.0104	0.0318	0.0350	0.0080	1.128	0.630
4th iteration	0.0012	0.0049	0.0069	0.0025	0.0121	0.0126	0.0019	1.042	0.625
5th iteration	0.0002	0.0016	0.0021	0.0006	0.0046	0.0045	0.0005	1.014	0.623
6th iteration	0.0000	0.0005	0.0007	0.0001	0.0017	0.0016	0.0001	1.005	0.623
7th iteration	0.0000	0.0002	0.0002	0.0000	0.0007	0.0006	0.0000	1.002	0.623

**Table 4:** Scenario 2 for Pat_1261 computations for enteric fever.

Initial Vector	0.75	0.75	0.58	A_i_(t + 1)	Signum Function
Weight	0.23	0.21	0.17	-	-
1st iteration	0.173	0.158	0.099	1.429	0.807
2nd iteration	0.129	0.118	0.057	1.305	0.787
3rd iteration	0.097	0.089	0.033	1.219	0.772
4th iteration	0.073	0.066	0.019	1.158	0.761
5th iteration	0.055	0.050	0.011	1.116	0.753
6th iteration	0.041	0.037	0.006	1.085	0.747

**Table 5:** Diagnostic Results for patient number Pat_1261.

Pat_1261	Actual Value	Expert Diagnosis	Computed Value	System Diagnosis
Malaria	0.91	Very High	0.7888	Yes
Enteric Fever	0.08	No	0.701	Yes
HIV AID	0.08	NO	0.795	No
UPUTI	0.08	No	0.6082	No
LWUTI	0.08	No	0.40	No
URTI	0.91	Very High	0.892	Yes
LRTI	0.08	No	0.9431	Yes
TB	0.08	No	0.7501	Yes
LASFVR	0.08	No	0.745	No
YELFVR	0.08	No	0.6226	No
DENFVR	0.08	No	0.7809	No

**Table 6:** Performance Metrics of the classification of 11 diseases.

Disease	Accuracy	Precision	Recall	F1
Malaria	0.752	0.796	0.840	0.817
Enteric Fever	0.718	0.507	0.765	0.609
HIV AIDS	0.908	0.603	0.467	0.526
UPUTI	0.857	0.522	0.685	0.592
LWUTI	0.754	0.442	0.762	0.561
URUTI	0.840	0.732	0.274	0.399
LRUTI	0.861	0.544	0.641	0.589
TB	0.757	0.232	0.830	0.363
LASFVR	0.993	0.250	0.076	0.118
YELFVR	0.998	0.5	0.333	0.400
DENFVR	0.989	0.272	0.150	0.194

**Table 7:** Performance measure of the Results.

Disease	TP	FP	FN	TN	Total
Malaria	1370	351	261	483	2465
Enteric Fever	543	529	167	1226	2465
HIV/AIDS	126	83	144	2112	2465
UPUTI	256	235	118	1856	2465
LWUTI	387	485	121	1472	2465
URTI	131	48	347	1939	2465
LRTI	245	205	137	1878	2465
TB	170	563	35	1697	2465
LASFVR	1	3	12	2449	2465
YELFVR	1	1	2	2461	2465
DENFVR	3	8	17	2437	2465

**Table 8:** Results of Diagnosis per Disease.

Name of Disease	No. of Actual Diagnoses	No. of Predicted Diagnoses
Malaria	1631	1721
Enteric Fever	710	1072
HIV/AIDS	270	209
UPUTI	374	491
LWUTI	508	872
URTI	478	179
LRTI	382	450
TB	205	733
LASFVR	13	04
YELFVR	02	01
DENFVR	11	11

## Data Availability

The data presented in this study are available on request from the corresponding author.

## Appendix A. Symptom Rankings for Diseases

**Table A1:** Symptom rankings for Enteric Fever.

SN	ENFVR Symptoms	Pearson Rank	Kendall Rank	Spearman Rank	Mean
1	ABDPN	0.249	0.225	0.250	0.24
2	SWRFVR	0.227	0.217	0.236	0.23
3	HDACH	0.212	0.196	0.224	0.21
4	DIZ	0.186	0.206	0.226	0.21
5	NUS	0.185	0.192	0.211	0.20
6	DRH	0.201	0.178	0.191	0.19
7	LTG	0.167	0.158	0.174	0.17
8	CNST	0.151	0.167	0.178	0.17
9	INTBLEPRF	0.145	0.138	0.147	0.14
10	PERTN	0.125	0.128	0.136	0.13

**Table A2:** Symptom rankings for HIV/AIDS.

SN	HVAD Symptoms	Pearson Rank	Kendall Rank	Spearman Rank	Mean
1	GENRSH	0.419	0.430	0.443	0.43
2	SKNRSH	0.417	0.418	0.432	0.42
3	MUTUCR	0.383	0.330	0.341	0.35
4	LMPNDSWL	0.402	0.320	0.331	0.35
5	DRH	0.299	0.276	0.287	0.29
6	BDYICH	0.232	0.246	0.256	0.24
7	NGTSWT	0.239	0.196	0.207	0.21
8	SRTRT	0.192	0.210	0.218	0.21
9	FOLBRT	0.204	0.188	0.195	0.20
10	CGHDRY	0.195	0.169	0.180	0.18
11	DRYCGH	0.160	0.142	0.150	0.15
12	DRYCGH	0.146	0.133	0.141	0.14
13	LTG	0.121	0.087	0.093	0.10
14	DIZ	0.099	0.073	0.078	0.08
15	LWGDFVR	0.079	0.081	0.088	0.08

**Table A3:** Symptom rankings for Upper Urinary-Tract Infection (UPUTI).

SN	UPUTI Symptoms	Pearson Rank	Kendall Rank	Spearman Rank	Mean
1	PNFLURNTN	0.553	0.503	0.529	0.53
2	URNFQC	0.553	0.487	0.514	0.52
3	SPPBPN	0.509	0.479	0.506	0.50
4	CLDYURN	0.508	0.443	0.465	0.47
5	BLDYURN	0.244	0.238	0.247	0.24
6	ABDPN	0.216	0.222	0.243	0.23
7	UPBCKPN	0.196	0.211	0.223	0.21
8	BCKPN	0.177	0.189	0.203	0.19
9	NUS	0.144	0.151	0.163	0.15
10	SUDONFVR	0.124	0.125	0.135	0.13
11	HGPSFVR	0.127	0.120	0.131	0.13

**Table A4:** Symptom rankings for Lower Urinary-Tract Infection (LWUTI).

SN	LWUTI Symptoms	Pearson Rank	Kendall Rank	Spearman Rank	Mean
1	URNFQC	0.592	0.551	0.584	0.58
2	PNFLURNTN	0.589	0.554	0.584	0.58
3	SPPBPN	0.528	0.529	0.562	0.54
4	CLDYURN	0.487	0.465	0.493	0.48
5	BLDYURN	0.268	0.263	0.277	0.27
6	ABDPN	0.212	0.225	0.249	0.23
7	UPBCKPN	0.194	0.195	0.207	0.20
8	BCKPN	0.120	0.140	0.152	0.14
9	LWGDFVR	0.106	0.102	0.113	0.11

**Table A5:** Symptom rankings Upper Respiratory-Tract Infection (URTI).

SN	URTI Symptoms	Pearson Rank	Kendall Rank	Spearman Rank	Mean
1	CTRH	0.447	0.403	0.429	0.43
2	CGHDRY	0.407	0.414	0.444	0.42
3	SRTRT	0.314	0.327	0.346	0.33
4	DIFBRT	0.302	0.318	0.338	0.32
5	DRYCGH	0.314	0.300	0.322	0.31
6	DRYCGH	0.311	0.296	0.317	0.31
7	FOLBRT	0.222	0.246	0.260	0.24
8	MUTUCR	0.118	0.163	0.172	0.15
9	FTG	0.112	0.120	0.135	0.12
10	HDACH	0.102	0.105	0.120	0.11
11	LWGDFVR	0.057	0.076	0.083	0.07

**Table A6:** Symptom rankings for Lower Respiratory-Tract Infection.

SN	LRTI Symptoms	Pearson Rank	Kendall Rank	Spearman Rank	Mean
1	CGHDRY	0.524	0.480	0.513	0.51
2	DIFBRT	0.429	0.435	0.461	0.44
3	CHSPN	0.411	0.410	0.438	0.42
4	WHZ	0.339	0.340	0.355	0.34
5	CHSIND	0.321	0.329	0.344	0.33
6	DRYCGH	0.282	0.293	0.313	0.30
7	DRYCGH	0.278	0.282	0.301	0.29
8	LMPNDSWL	0.229	0.259	0.272	0.25
9	FOLBRT	0.235	0.254	0.268	0.25
10	NGTSWT	0.241	0.247	0.263	0.25
11	SRTRT	0.219	0.254	0.268	0.25
12	CTRH	0.209	0.219	0.235	0.22
13	MUTUCR	0.177	0.213	0.223	0.20
14	LWGDFVR	0.078	0.099	0.108	0.09

**Table A7:** Symptom rankings for Tuberculosis (TB).

SN	TB Symptoms	Pearson Rank	Kendall Rank	Spearman Rank	Mean
1	CGHDRY	0.481	0.419	0.443	0.45
2	NGTSWT	0.503	0.392	0.409	0.43
3	CHSPN	0.390	0.373	0.391	0.38
4	DIFBRT	0.310	0.334	0.348	0.33
5	LMPNDSWL	0.345	0.315	0.327	0.33
6	MUTUCR	0.269	0.271	0.279	0.27
7	FOLBRT	0.275	0.261	0.270	0.27
8	CHSIND	0.251	0.266	0.273	0.26
9	WHZ	0.244	0.265	0.272	0.26
10	SRTRT	0.215	0.233	0.243	0.23
11	DRYCGH	0.233	0.221	0.233	0.23
12	DRYCGH	0.223	0.214	0.226	0.22
13	LWGDFVR	0.218	0.185	0.200	0.20

**Table A8:** Symptom rankings for Laser Fever (LASFVR).

SN	LASFVR Symptoms	Pearson Rank	Kendall Rank	Spearman Rank	Mean
1	REDEYE	0.216	0.181	0.182	0.19
2	REDEYEFCTNG	0.247	0.165	0.166	0.19
3	SENLHT	0.201	0.099	0.100	0.13
4	PNBHEYE	0.150	0.106	0.107	0.12
5	JNTSWL	0.113	0.114	0.115	0.11
6	PERTN	0.120	0.107	0.108	0.11
7	BLDYURN	0.089	0.118	0.119	0.11
8	SHK	0.131	0.087	0.087	0.10
9	SRTRT	0.111	0.082	0.084	0.09
10	UPBCKPN	0.070	0.089	0.091	0.08
11	SUDONFVR	0.087	0.076	0.081	0.08
12	BLDN	0.086	0.076	0.076	0.08

**Table A9:** Symptom rankings for Yellow Fever (YELFVR).

SN	YELFVR Symptoms	Pearson Rank	Kendall Rank	Spearman Rank	Mean
1	PERTN	0.103	0.093	0.094	0.10
2	JNTSWL	0.072	0.081	0.082	0.08
3	DRH	0.051	0.080	0.082	0.07
4	BDYICH	0.068	0.071	0.072	0.07
5	SHK	0.061	0.073	0.074	0.07
6	LMPNDSWL	0.062	0.071	0.072	0.07
7	SENLHT	0.093	0.052	0.053	0.07
8	GENRSH	0.073	0.062	0.063	0.07
9	INTBLEPRF	0.055	0.069	0.070	0.06
10	BLDN	0.063	0.063	0.064	0.06
11	HGPSFVR	0.038	0.068	0.072	0.06

**Table A10:** Symptom rankings for DENGUE Fever (DENFVR).

SN	DENFVR Symptoms	Pearson Rank	Kendall Rank	Spearman Rank	Mean
1	REDEYE	0.054	0.130	0.131	0.10
2	REDEYEFCTNG	0.066	0.101	0.101	0.09
3	HGGDFVR	0.078	0.083	0.089	0.08
4	UPBCKPN	0.053	0.090	0.092	0.08
5	SENLHT	0.048	0.088	0.089	0.08
6	SKNRSH	0.055	0.079	0.080	0.07
7	BCKPN	0.053	0.077	0.081	0.07
8	PNBHEYE	0.059	0.075	0.076	0.07
9	SHK	0.072	0.069	0.070	0.07
10	PERTN	0.052	0.059	0.059	0.06
11	LMPNDSWL	0.047	0.060	0.062	0.06
12	INTBLEPRF	0.056	0.055	0.056	0.06
13	DIZ	0.040	0.061	0.065	0.06
14	ABDPN	0.047	0.056	0.060	0.05
15	GENRSH	0.031	0.065	0.066	0.05
16	CHSPN	0.038	0.059	0.061	0.05
17	HGPSFVR	0.025	0.062	0.066	0.05
18	FTG	0.037	0.047	0.052	0.05
19	DIFBRT	0.030	0.051	0.052	0.04
20	MSCBDYPN	0.042	0.043	0.046	0.04
21	BLDYURN	0.022	0.054	0.054	0.04
22	GENBDYPN	0.039	0.042	0.046	0.04

## Appendix B. Symptoms, Diseases and Their Meaning

**Table A11:** Acronyms and their Meanings for the Symptoms and Diseases.

Label	Symptom	Meaning	Label	Symptom	Meaning	Label	Symptom	Meaning
T1	ABDPN	abdominal pain	T21	SWRFVR	stepwise rise fever	T41	REDEYE	Red eye
T2	BCKPN	back pain	T22	SUDONFVR	sudden onset fever	T42	REDEYEFCTNG	red eye face and tongue
T3	BITAIM	bitter test in mouth	T23	LWGDFVR	low-grade fever	T43	SENLHT	sensitivity to light
T4	BLDN	bleeding from any sight	T24	FOLBRT	foul breathe	T44	SHK	shock
T5	BLDYURN	bloody urine	T25	BDYICH	body itching	T45	SKNRSH	skin rash
T6	CTRH	Catarrh	T26	GENBDYPN	generalized body pain	T46	SRTRT	sore throat
T7	CHSIND	chest indraw	T27	GENRSH	generalized rashes	T47	SPPBPN	suprapubic pains
T8	CHSPN	chest pain	T28	HDACH	Headache	T48	URNFQC	urinary frequency
T9	CHLNRIG	chills and rigours	T29	INTBLEPRF	intestinal bleeding and perforation	T49	VMT	vomiting
T10	CLDYURN	cloudy urine	T30	JNTSWL	joint swelling	T50	WHZ	wheeze
T11	CNST	constipation	T31	LTG	lethargy			
T12	CGHDRY	cough initial dry	T32	LMPNDSWL	lymph node swelling	D1	MAL	malaria
T13	DRH	diarrhoea	T33	MSCBDYPN	muscle and body pain	D2	ENFVR	enteric ever
T14	DIFBRT	difficulty breathing	T34	MUTUCR	mouth ulcer	D3	HVAD	HIV/AIDS
T15	DIZ	dizziness	T35	NUS	nausea	D4	UPUTI	upper urinary-tract infection
T16	DRYCGH	dry cough	T36	NGTSWT	night sweats	D5	LWUTI	lower urinary-tract infection
T17	FTG	Fatigue	T37	PNBHEYE	pain behind eye	D6	URTI	upper respiratory-tract infection
T18	FVR	Fever	T38	UPBCKPN	upper back pain	D7	LRTI	lower respiratory-tract infection
T19	HGPSFVR	high persistent fever	T39	PNFLURNTN	painful urination	D8	TB	tuberculosis
T20	HGGDFVR	high grade fever	T40	PERTN	peritonitis	D9	LASFVR	Laser Fever
						D10	YELFVR	Yellow Fever
						D11	DENFVR	Dengue Fever

## Appendix C. Sample Results

**Table A12:** Malaria(MAL) to Upper Urinary Tract Infection(UPUTI) Results.

Pat_No	Mal_Data	Diagnosis	FCM_Mal	Diagnosis	Ent_Data	Diagnosis	FCM_Ent	Diagnosis	HivAd_Data	Diagnosis	FCM_HivAd	Diagnosis	Uputi_data	Diagnosis	FCM_Uputi	Diagnosis
1261	0.91	MAL	0.7888	MAL	0.08	No	0.701	ENTFV	0.08	NO	0.795	No	0.08	No	0.6082	No
1377	0.58	MAL	0.6727	MAL	0.08	No	0.3603	No	0.08	NO	0.4223	No	0.08	No	0.4782	No
1459	0.08	No	0.2832	No	0.08	No	0.3212	No	0.08	NO	0.4835	No	0.08	No	0.5132	No
1480	0.58	MAL	0.9987	MAL	0.58	ENTFV	0.5524	ENTFV	0.08	NO	0.3832	No	0.08	No	0.516	No
151	0.58	MAL	0.6151	MAL	0.08	No	0.4334	No	0.08	NO	0.3968	No	0.08	No	0.5173	No
1594	0.58	MAL	0.6419	MAL	0.08	No	0.3212	No	0.08	NO	0.3968	No	0.08	No	0.434	No
1728	0.58	MAL	0.7083	MAL	0.58	ENTFV	0.5474	ENTFV	0.58	HVAD	0.7915	No	0.08	No	0.5277	No
1902	0.75	MAL	0.9452	MAL	0.75	ENTFV	0.6157	ENTFV	0.08	NO	0.4162	No	0.08	No	0.5528	No
2019	0.08	No	0.3822	No	0.08	No	0.4004	No	0.08	NO	0.4138	No	0.08	No	0.6419	No
2054	0.58	MAL	0.6181	MAL	0.91	ENTFV	0.8359	ENTFV	0.08	NO	0.4699	No	0.08	No	0.5927	No
2055	0.75	MAL	0.7652	MAL	0.41	ENTFV	0.8877	ENTFV	0.08	NO	0.8364	HVAD	0.08	No	0.797	No
2106	0.58	MAL	0.6134	MAL	0.75	ENTFV	0.9595	ENTFV	0.08	NO	0.5586	No	0.41	UPUTI	0.978	UPUTI
2183	0.41	MAL	0.8928	MAL	0.41	ENTFV	0.6858	ENTFV	0.08	NO	0.8977	HVAD	0.08	No	0.6686	No
2365	0.25	MAL	0.4666	No	0.58	ENTFV	0.6031	ENTFV	0.08	NO	0.3968	No	0.08	No	0.5881	No
2417	0.08	No	0.6344	MAL	0.08	No	0.638	ENTFV	0.08	NO	0.4232	No	0.08	No	0.5226	No
2622	0.75	MAL	0.7044	MAL	0.75	ENTFV	0.6647	ENTFV	0.08	NO	0.86	HVAD	0.08	No	0.564	No
2846	0.75	MAL	0.6576	MAL	0.41	ENTFV	0.6022	ENTFV	0.08	NO	0.4597	No	0.08	No	0.4835	No
2958	0.75	MAL	0.8948	MAL	0.75	ENTFV	0.8347	ENTFV	0.08	NO	0.5859	No	0.08	No	0.7519	No
2983	0.08	No	0.4082	No	0.08	No	0.467	No	0.08	NO	0.4232	No	0.08	No	0.5054	No
3056	0.91	MAL	0.7035	MAL	0.08	No	0.3212	No	0.08	NO	0.5137	No	0.08	No	0.434	No
3237	0.41	MAL	0.7368	MAL	0.08	No	0.4719	No	0.08	NO	0.4667	No	0.08	No	0.5347	No
3499	0.75	MAL	0.7079	MAL	0.08	No	0.4466	No	0.08	NO	0.4519	No	0.08	No	0.434	No
350	0.58	MAL	0.6704	MAL	0.08	No	0.5976	ENTFV	0.08	NO	0.6519	No	0.25	UPUTI	0.969	UPUTI
3562	0.08	No	0.4119	No	0.08	No	0.4664	No	0.08	NO	0.3832	No	0.08	No	0.5594	No
3582	0.58	MAL	0.7343	MAL	0.58	ENTFV	0.5578	ENTFV	0.08	NO	0.4232	No	0.08	No	0.4769	No
3624	0.75	MAL	0.6654	MAL	0.91	ENTFV	0.797	ENTFV	0.08	NO	0.4718	No	0.08	No	0.8133	UPUTI
3683	0.41	MAL	0.6176	MAL	0.58	ENTFV	0.852	ENTFV	0.75	HVAD	0.9717	HVAD	0.58	UPUTI	0.9	UPUTI
3737	0.08	No	0.5208	No	0.08	No	0.4664	No	0.08	NO	0.3832	No	0.08	No	0.5594	No
3738	0.08	No	0.5208	No	0.08	No	0.4664	No	0.08	NO	0.3832	No	0.08	No	0.5594	No
3886	0.58	MAL	0.7871	MAL	0.58	ENTFV	0.4423	No	0.08	NO	0.3832	No	0.08	No	0.4769	No
3890	0.58	MAL	0.7079	MAL	0.58	ENTFV	0.5474	ENTFV	0.08	NO	0.5251	No	0.08	No	0.5693	No
4221	0.75	MAL	0.8449	MAL	0.08	No	0.4004	No	0.08	NO	0.4232	No	0.08	No	0.5099	No
4490	0.08	No	0.6315	MAL	0.08	No	0.4434	No	0.58	HVAD	0.8481	HVAD	0.08	No	0.434	No
4644	0.58	MAL	0.7216	MAL	0.58	ENTFV	0.6535	ENTFV	0.08	NO	0.3832	No	0.08	No	0.4835	No
4742	0.41	MAL	0.6811	MAL	0.08	No	0.3212	No	0.08	NO	0.3832	No	0.08	No	0.4769	No UPUTI
479	0.25	MAL	0.7379	MAL	0.58	ENTFV	0.5875	ENTFV	0.08	NO	0.4096	No	0.08	No	0.4769	No
797	0.08	No	0.5769	No	0.75	ENTFV	0.6548	ENTFV	0.08	NO	0.7837	No	0.08	No	0.7335	No UPUTI
802	0.08	No	0.5769	No	0.75	ENTFV	0.6548	ENTFV	0.08	NO	0.7837	No	0.08	No	0.7335	No
1224	0.91	MAL	0.7292	MAL	0.91	ENTFV	0.8821	ENTFV	0.08	NO	0.6275	No	0.08	No	0.8373	UPUTI
129	0.08	No	0.2832	No	0.08	No	0.3212	No	0.08	NO	0.3832	No	0.08	No	0.434	No
1364	0.08	No	0.5451	No	0.75	ENTFV	0.5888	ENTFV	0.75	HVAD	0.556	No	0.08	No	0.499	No
1374	0.08	No	0.6869	MAL	0.58	ENTFV	0.5507	ENTFV	0.58	HVAD	0.5179	No	0.08	No	0.4782	No
1584	0.08	No	0.2832	No	0.08	No	0.3212	No	0.08	NO	0.3832	No	0.08	No	0.434	No
174	0.58	MAL	0.6079	MAL	0.91	ENTFV	0.8052	ENTFV	0.08	NO	0.3968	No	0.08	No	0.5985	No
183	0.08	No	0..8079	MAL	0.08	No	0.8052	ENTFV	0.08	NO	0.3968	No	0.08	No	0.5985	No
2023	0.75	MAL	0.8929	MAL	0.08	No	0.3926	No	0.08	NO	0.4727	No	0.08	No	0.434	No
2024	0.75	MAL	0.7779	MAL	0.41	ENTFV	0.5887	ENTFV	0.08	NO	0.3968	No	0.58	UPUTI	0.782	No UPUTI
2045	0.91	MAL	0.8546	MAL	0.41	ENTFV	0.7918	ENTFV	0.08	NO	0.4426	No	0.08	No	0.7668	No UPUTI
2088	0.41	MAL	0.5897	No	0.08	No	0.4181	No	0.08	NO	0.5935	No	0.08	No	0.4731	No UPUTI

**Table A13:** Lower Unrinary Tract Infection(LWUTI) to Tuberculosis(TB) Results.

Pat_No	Lwuti_Data	Diagnosis	FCM_Lwuti	Diagnosis	Urti_Data	Diagnosis	FCM_Urti	Diagnosis	Lrti_Data	Diagnosis	FCM_Lrti	Diagnosis	TB_Data	Diagnosis	FCM_TB	Diagnosis
1261	0.08	No	0.40	No	0.91	URTI	0.892	URTI	0.08	No	0.9434	LRTI	0.08	No TB	0.7501	TB
1377	0.08	No	0.79	No	0.58	URTI	0.5492	No	0.08	No	0.5669	No	0.08	No TB	0.3843	No TB
1459	0.75	LWUTI	0.864	LWUTI	0.08	No	0.2505	No	0.08	No	0.462	No	0.08	No TB	0.3571	No TB
1480	0.08	No	0.51	No	0.08	No	0.2783	No	0.08	No	0.428	No	0.08	No TB	0.3112	No TB
151	0.08	No	0.51	No	0.08	No	0.2403	No	0.08	No	0.428	No	0.08	No TB	0.3112	No TB
1594	0.08	No	0.49	No	0.08	No	0.3754	No	0.08	No	0.5159	No	0.08	No TB	0.3452	No TB
1728	0.08	No	0.49	No	0.08	No	0.469	No	0.08	No	0.6966	No	0.08	No TB	0.5787	TB
1902	0.08	No	0.65	No	0.08	No	0.2816	No	0.08	No	0.428	No	0.08	No TB	0.3112	No TB
2019	0.08	No	0.981	LWUTI	0.08	No	0.293	No	0.08	No	0.5147	No	0.08	No TB	0.3877	No TB
2054	0.08	No	0.77	No	0.08	No	0.3162	No	0.08	No	0.428	No	0.08	No TB	0.3112	No TB
2055	0.08	No	0.892	LWUTI	0.08	No	0.6618	No	0.08	No	0.7454	No	0.08	No TB	0.6142	TB
2106	0.58	LWUTI	0.921	LWUTI	0.08	No	0.4268	No	0.08	No	0.553	No	0.08	No TB	0.4432	No TB
2183	0.08	No	0.864	LWUTI	0.41	URTI	0.871	URTI	0.91	LRTI	0.9204	LRTI	0.91	TB	0.9922	TB
2365	0.08	No	0.543	LWUTI	0.08	No	0.2335	No	0.08	No	0.4433	No	0.08	No TB	0.3452	No TB
2417	0.08	No	0.671	No	0.08	No	0.297	No	0.08	No	0.428	No	0.08	No TB	0.3112	No TB
2622	0.08	No	0.783	No	0.75	URTI	0.8249	URTI	0.08	No	0.8363	LRTI	0.75	TB	0.8226	TB
2846	0.08	No	0.657	No	0.08	No	0.3162	No	0.08	No	0.4705	No	0.08	No TB	0.3673	No TB
2958	0.08	No	0.846	LWUTI	0.08	No	0.3502	No	0.08	No	0.4858	No	0.08	No TB	0.3911	No TB
2983	0.08	No	0.73	No	0.08	No	0.336	No	0.08	No	0.6117	No	0.08	No TB	0.4531	No TB
3056	0.08	No	0.567	No	0.08	No	0.2743	No	0.08	No	0.604	No	0.08	No TB	0.5653	TB
3237	0.08	No	0.721	No	0.08	No	0.3834	No	0.08	No	0.5695	No	0.08	No TB	0.433	No TB
3499	0.08	No	0.432	No	0.08	No	0.2783	No	0.08	No	0.4705	No	0.08	No TB	0.3843	No TB
350	0.08	No	0.965	LWUTI	0.08	No	0.5196	No	0.08	No	0.8165	LRTI	0.08	No TB	0.8177	TB
3562	0.08	No	0.6545	No	0.08	No	0.2216	No	0.08	No	0.428	No	0.08	No TB	0.3112	No TB
3582	0.08	No	0.4323	No	0.08	No	0.2929	No	0.08	No	0.473	No	0.08	No TB	0.4112	No TB
3624	0.08	No	0.8542	LWUTI	0.41	URTI	0.8058	URTI	0.41	LRTI	0.9551	LRTI	0.08	No TB	0.7166	TB
3683	0.58	LWUTI	0.879	LWUTI	0.41	URTI	0.7496	No	0.41	LRTI	0.8036	LRTI	0.58	TB	0.8128	TB
3737	0.08	No	0.65	No	0.08	No	0.2216	No	0.08	No	0.428	No	0.08	No TB	0.3112	No TB
3738	0.08	No	0.435	No	0.08	No	0.2216	No	0.08	No	0.428	No	0.08	No TB	0.3112	No TB
3886	0.08	No	0.567	No	0.08	No	0.2216	No	0.08	No	0.428	No	0.08	No TB	0.3112	No TB
3890	0.08	No	0.677	No	0.08	No	0.2216	No	0.08	No	0.428	No	0.08	No TB	0.3112	No TB
4221	0.08	No	0.905	LWUTI	0.08	No	0.2962	No	0.08	No	0.473	No	0.08	No TB	0.4112	No TB
4490	0.08	No	0.543	No	0.08	No	0.5862	No	0.08	No	0.7349	No	0.08	No TB	0.5414	TB
4644	0.08	No	0.432	No	0.08	No	0.2579	No	0.08	No	0.428	No	0.08	No TB	0.3112	No TB
4742	0.08	No	0.432	No	0.08	No	0.2216	No	0.08	No	0.428	No	0.08	No TB	0.3112	No TB
479	0.08	No	0.785	No	0.08	No	0.3206	No	0.08	No	0.4577	No	0.08	No TB	0.3772	No TB
797	0.08	No	0.965	LWUTI	0.08	No	0.2579	No	0.08	No	0.428	No	0.08	No TB	0.3112	No TB
802	0.08	No	0.8965	LWUTI	0.08	No	0.2579	No	0.08	No	0.428	No	0.08	No TB	0.3112	No TB
1224	0.08	No	0.971	LWUTI	0.08	No	0.3757	No	0.08	No	0.428	No	0.08	No TB	0.3112	No TB
129	0.08	No	0.77	No	0.08	No	0.2216	No	0.08	No	0.428	No	0.08	No TB	0.3112	No TB
1364	0.08	No	0.69	No	0.08	No	0.2698	No	0.08	No	0.4433	No	0.08	No TB	0.3452	No TB
1374	0.08	No	0.49	No	0.08	No	0.3094	No	0.08	No	0.4433	No	0.08	No TB	0.3452	No TB
1584	0.08	No	0.647	No	0.08	No	0.2216	No	0.08	No	0.428	No	0.08	No TB	0.3112	No TB
174	0.08	No	0.919	LWUT	0.08	No	0.2975	No	0.08	No	0.428	No	0.08	No TB	0.3112	No TB
183	0.08	No	0.935	LWUT	0.08	No	0.2975	No	0.08	No	0.428	No	0.08	No TB	0.3112	No TB
2023	0.08	No	0.431	No	0.08	No	0.3861	No	0.08	No	0.5567	No	0.08	No TB	0.4531	No TB
2024	0.41	LWUTI	0.8654	LWUTI	0.08	No	0.2522	No	0.08	No	0.5147	No	0.08	No TB	0.4098	No TB
2045	0.08	No	0.874	LWUTI	0.08	No	0.3162	No	0.08	No	0.428	No	0.08	No TB	0.3112	No TB
2088	0.08	No	0.95	LWUT	0.75	URTI	0.8871	URTI	0.41	LRTI	0.878	LRTI	0.08	No TB	0.6539	TB

**Table A14:** Lassa Fever(LASFVR) to Dengue Fever(DENFVR) Results.

Pat_No	Lasfv_Data	Diagnosis	FCM_lasfv	Diagnosis	Yelfv_Data	Diagnosis	FCM_Yelfv	Diagnosis	Denfv_Data	Diagnosis	FCM_Denfv	Diagnosis
1261	0.08	No	0.7451	No	0.08	No	0.6226	No	0.08	No	0.7809	No
1377	0.08	No	0.6448	No	0.08	No	0.5926	No	0.08	No	0.6509	No
1459	0.08	No	0.6675	No	0.08	No	0.5943	No	0.08	No	0.642	No
1480	0.08	No	0.6312	No	0.08	No	0.6022	No	0.08	No	0.7023	No
151	0.08	No	0.6448	No	0.08	No	0.5926	No	0.08	No	0.6696	No
1594	0.08	No	0.6312	No	0.08	No	0.5824	No	0.08	No	0.6288	No
1728	0.08	No	0.6601	No	0.08	No	0.6045	No	0.08	No	0.6713	No
1902	0.08	No	0.6312	No	0.08	No	0.6022	No	0.08	No	0.6936	No
2019	0.08	No	0.6939	No	0.08	No	0.6088	No	0.08	No	0.7212	No
2054	0.08	No	0.6448	No	0.08	No	0.6345	No	0.08	No	0.7575	No
2055	0.08	No	0.9334	LASFVR	0.08	No	0.9036	No	0.08	No	1.581	No
2106	0.08	No	0.6932	No	0.08	No	0.6413	No	0.08	No	0.829	No
2183	0.08	No	0.8012	No	0.08	No	0.626	No	0.08	No	0.8661	No
2365	0.08	No	0.6312	No	0.08	No	0.5824	No	0.08	No	0.6623	No
2417	0.08	No	0.6312	No	0.08	No	0.5824	No	0.08	No	0.6758	No
2622	0.08	No	0.6712	No	0.08	No	0.6355	No	0.08	No	0.7703	No
2846	0.08	No	0.6312	No	0.08	No	0.5943	No	0.08	No	0.7091	No
2958	0.08	No	0.6856	No	0.08	No	0.648	No	0.08	No	0.6957	No
2983	0.08	No	0.6712	No	0.08	No	0.6124	No	0.08	No	0.724	No
3056	0.08	No	0.6448	No	0.08	No	0.5926	No	0.08	No	0.682	No
3237	0.08	No	0.6652	No	0.08	No	0.6124	No	0.08	No	0.7393	No
3499	0.08	No	0.6312	No	0.08	No	0.5824	No	0.08	No	0.65	No
350	0.08	No	0.756	No	0.08	No	0.661	No	0.08	No	0.8382	No
3562	0.08	No	0.6312	No	0.08	No	0.5824	No	0.08	No	0.6453	No
3582	0.08	No	0.6576	No	0.08	No	0.6418	No	0.08	No	0.6651	No
3624	0.08	No	0.7448	No	0.08	No	0.6124	No	0.08	No	0.8425	No
3683	0.08	No	0.6894	No	0.08	No	0.6688	No	0.08	No	0.7573	No
3737	0.08	No	0.6312	No	0.08	No	0.5824	No	0.08	No	0.6453	No
3738	0.08	No	0.6312	No	0.08	No	0.5824	No	0.08	No	0.6453	No
3886	0.08	No	0.6312	No	0.08	No	0.6022	No	0.08	No	0.6849	No
3890	0.08	No	0.6576	No	0.08	No	0.6253	No	0.08	No	0.7014	No
4221	0.08	No	0.6312	No	0.08	No	0.5824	No	0.08	No	0.675	No
4490	0.08	No	0.6312	No	0.08	No	0.6276	No	0.08	No	0.6543	No
4644	0.08	No	0.6576	No	0.08	No	0.6022	No	0.08	No	0.6552	No
4742	0.08	No	0.6576	No	0.08	No	0.5824	No	0.08	No	0.6288	No
479	0.08	No	0.6312	No	0.08	No	0.6022	No	0.08	No	0.6882	No
797	0.08	No	0.6312	No	0.08	No	0.6405	No	0.08	No	0.682	No
802	0.08	No	0.6312	No	0.08	No	0.6405	No	0.08	No	0.682	No
1224	0.08	No	0.6848	No	0.08	No	0.6695	No	0.08	No	0.8029	No
129	0.08	No	0.6312	No	0.08	No	0.5824	No	0.08	No	0.6288	No
1364	0.08	No	0.6448	No	0.08	No	0.6022	No	0.08	No	0.6589	No
1374	0.08	No	0.6448	No	0.08	No	0.5926	No	0.08	No	0.6674	No
1584	0.08	No	0.6312	No	0.08	No	0.5824	No	0.08	No	0.6288	No
174	0.08	No	0.6312	No	0.08	No	0.5824	No	0.08	No	0.7273	No
183	0.08	No	0.6312	No	0.08	No	0.5824	No	0.08	No	0.7273	No
2023	0.08	No	0.6312	No	0.08	No	0.5824	No	0.08	No	0.6543	No
2024	0.08	No	0.6576	No	0.08	No	0.6226	No	0.08	No	0.738	No
2045	0.08	No	0.6312	No	0.08	No	0.6124	No	0.08	No	0.7846	No
2088	0.08	No	0.6312	No	0.08	No	0.5824	No	0.08	No	0.6538	No
